# 53BP1 contributes to a robust genomic stability in human fibroblasts

**DOI:** 10.18632/aging.100381

**Published:** 2011-09-08

**Authors:** Lauren S. Fink, Michaela Roell, Emanuela Caiazza, Chad Lerner, Thomas Stamato, Silvana Hrelia, Antonello Lorenzini, Christian Sell

**Affiliations:** ^1^ Department of Pathology, Drexel University College of Medicine, Philadelphia, PA 19102, USA; ^2^ Department of Biochemistry, “G. Moruzzi” University of Bologna, Bologna, Italy; ^3^ Lankenau Institute for Medical Research, Wynnewood, PA 19096, USA; ^4^ Department of Cancer Biology, Fox Chase Cancer Center, Philadelphia, PA 19111, USA

**Keywords:** 53BP1, micronuclei, fibroblasts, mouse, human, stability, genome

## Abstract

Faithful repair of damaged DNA is a crucial process in maintaining cell viability and function. A multitude of factors and pathways guides this process and includes repair proteins and cell cycle checkpoint factors. Differences in the maintenance of genomic processes are one feature that may contribute to species-specific differences in lifespan. We predicted that 53BP1, a key transducer of the DNA damage response and cell cycle checkpoint control, is highly involved in maintaining genomic stability and may function differently in cells from different species. We demonstrate a difference in the levels and recruitment of 53BP1 in mouse and human cells following DNA damage. In addition, we show that unresolved DNA damage persists more in mouse cells than in human cells, as evidenced by increased numbers of micronuclei. The difference in micronuclei seems to be related to the levels of 53BP1 present in cells. Finally, we present evidence that unresolved DNA damage correlates with species lifespan. Taken together, these studies suggest a link between recruitment of 53BP1, resolution of DNA damage, and increased species lifespan.

## INTRODUCTION

Human cells exhibit a greatly enhanced stringency for growth control and greater genomic stability when compared to rodent cells [1]. This higher stringency is thought to underlie the relative resistance of humans to malignancies. For example, it has been estimated that the difference in the spontaneous immortalization rate between rodents and human cells is 10^−5^ to 10^−6^ compared to 10^−9^ to 10^−10^[2]. This difference is reflected experimentally in studies which introduced active oncogenes to drive malignant transformation in primary cells. Rodent cells required a single oncogenic mutation coupled with abrogation of the major cell cycle checkpoint regulator p53, while human cells required the addition of multiple oncogenic mutations targeting several intracellular pathways critical to cell cycle progression and survival, which include p53, pRb, and the Ras/MAPK pathway as well as cellular phosphatases [3]. Substantial differences have been found at the cellular level that may contribute to the species-specific differences in genomic stability between rodent and human cells. For example, telomere biology differs significantly between rodent and human cells [4], and the spindle checkpoint has been found to be much less stable in rodent cells [5,6]. In addition, there is variation in essential DNA repair proteins such the Ku70/80 heterodimer and DNA end binding, although absolute DNA repair rates appear to be similar in rodents and humans as assessed by standard assays [7].

We have sought to further understand the differences in genomic stability between rodent and human cells by examining the formation of micronuclei, small membrane bound DNA fragments that form as a result of unresolved DNA damage, and the accumulation of 53BP1 into DNA damage foci. We find that significant differences exist in both spontaneous and DNA damage-induced rates of micronuclei formation as well as the accumulation of 53BP1.

## RESULTS

### DNA repair and cell cycle control are linked to micronuclei

**Figure 1:**
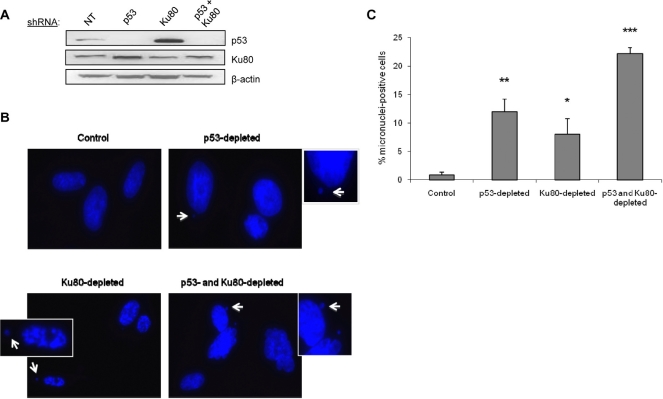
Depletion of p53 or Ku80 increases the rate of spontaneous micronuclei formation in WI38 human fibroblasts WI38 fibroblasts were infected with p53, Ku80, or both shRNA constructs and selected as described previously in Materials and Methods. Seven days after the infection, cells were fixed and stained with DAPI to visualize nuclear structure. (**A**) Representative Western blot showing p53 and Ku80 protein levels following shRNA infection. Ku80 knockdown is 30% and Ku80-depleted WI38 cells display a 3.9-fold increase in p53 by densitometric analysis. β-actin is shown as a protein loading control. NT=non-transfected cells. (**B**) Representative DAPI images for each cell type. Examples of micronuclei are marked with white arrows, enlarged and displayed as insets with white borders. (**C**) Quantitation of micronuclei, represented by the percent of cells containing at least one micronucleus. Data are pooled from two independent experiments, where at least 400 cells from each condition were counted per experiment, and standard deviation is included. Statistics determined using Student's T test are indicated by: *P<0.05; **P<0.01; ***P<0.001.

Due to its involvement in multiple genomic functions, we postulated that the Ku heterodimer functions in both DNA repair and in facilitating cell cycle arrest to ensure faithful repair has been completed. To begin to address this possibility, we used a targeted knockdown approach to reduce the levels of Ku80 and p53 in WI38 fibroblasts using shRNA vectors that we have found to be effective for reducing Ku80 and p53 levels in these cells (Figure 1A, Supplemental Figure 1) [8]. As a measure of genomic integrity and proper DNA repair, we examined the formation of micronuclei, which are known to form when cells enter mitosis with unresolved chromosomal damage. We predicted that decreasing Ku levels would increase micronuclei formation in human cells. As predicted, more than 8-fold more cells with micronuclei were found in Ku80-depleted fibroblasts compared with control fibroblasts (Figure 1B,C). Interestingly, we found that targeting Ku80 resulted in an increased number of cells with micronuclei even in the absence of exogenous DNA damage (Figure 1B,C). As a positive control, we targeted p53 using shRNA, and the p53 knockdown cells also contained a higher percentage of micronuclei-positive cells confirming that genomic instability can arise upon inactivation of p53 pathways (Figure 1A-C) [9]. As would be predicted, fibroblasts deficient in both p53 and Ku80 contained the most micronuclei-positive cells: 22% positive compared to 12% positive and 8% positive in p53-depleted and Ku80-depleted, respectively (Figure 1B,C).

**Figure 2:**
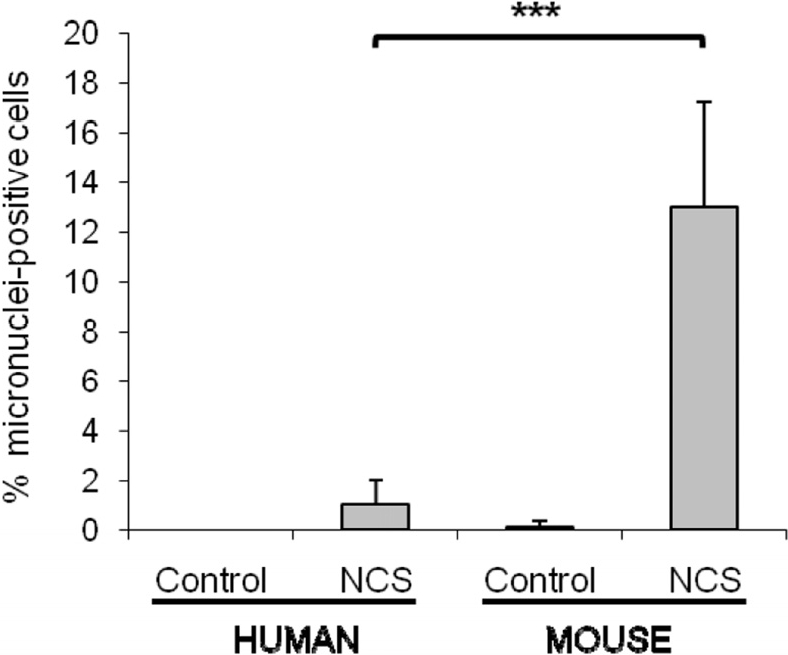
Mouse cells have a significantly higher number of micronuclei in comparison with human cells after the same genotoxic damage. WI38 human and HME mouse fibroblasts were treated with 1.5μg/mL neocarzinostatin (NCS) for 2 hours in serum free medium, then cells were washed and fed with regular growth media (10% FBS). Micronuclei were scored after 72 hours. For both species, fibroblasts were both of embryonic or adult origin. The percent of cells containing at least one micronucleus was scored on a minimum of 400 cells, and data are average of 4 and 8 independent experiments for human and mouse, respectively. Significance was calculated with Student's T test (***P<0.001). Non-damaged WI38 and HME cells were included as controls.

Because we had previously noted that rodent cells express significantly lower levels of Ku80 than human cells [7], we examined micronuclei formation in mouse cells and compared this with human cells. We found that mouse cultures have a higher percentage of micronuclei-positive cells than human cultures following treatment with the DNA-damaging agent neocarzinostatin (Figure 2).

### Mouse and human fibroblasts exhibit differential responses to DNA damage

**Figure 3:**
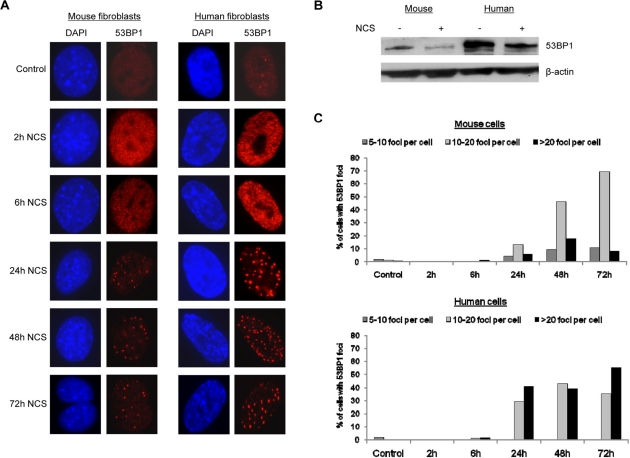
Human cells contain more 53BP1 foci following DNA damage (**A**) Mouse fibroblasts and human fibroblasts were seeded onto coverslips and treated with 1.5 ug/mL neocarzinostatin for 2 hours. Cells were fixed either right after 2 hours of damage or at 6, 24, 48, or 72 hours following damage and stained for 53BP1 foci. At least 400 cells per time point were counted, and untreated mouse and human fibroblasts were included as controls. Images were analyzed using Slidebook software equipped with deconvolution capabilities. (**B**) Western blot analysis of 53BP1 levels in mouse and human cells. Mouse and human cells were subjected to DNA damage as in A and allowed to recover for 72 hours when 53BP1 levels began to decline by immunofluorescence. Total protein was extracted and examined for the relative level of 53BP1 compared to untreated controls. β-actin was included as a loading control. (**C**) Quantification of 53BP1 foci formation in mouse and human cells is presented. Cells scored for 53BP1 foci in A were grouped according to the number of foci per cell: 5-10 foci, 10-20 foci, or >20 foci per cell.

During the evaluation of micronuclei, we examined 53BP1 accumulation by immunofluorescence and noted a significant difference between mouse and human cells. After induction of DNA damage with neocarzinostatin, human fibroblasts contained more visible 53BP1 foci than mouse fibroblasts (compare human cells in right panel to mouse cells in left panel in Figure 3A). This difference in micronuclei after damage was confirmed by Western blot analysis using an antibody that was raised against an epitope of 53BP1 that is conserved between mice and humans (Figure 3B). The number of 53BP1 foci per cell was quantified, and although both mouse and human fibroblasts displayed an increase in 53BP1 focus formation after DNA damage induction, a higher percentage of human fibroblasts contained greater than twenty 53BP1 foci per cell by 72 hours following induction of DNA damage (Figure 3C). These findings suggest that human fibroblasts display a more robust activation or upregulation of 53BP1 in response to DNA damage.

### Reducing 53BP1 in human cells changes their response to damage

**Figure 4:**
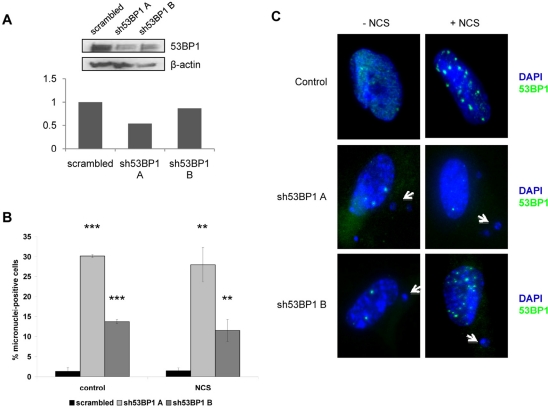
53BP1 depletion increases chromosomal instability in WI38 human fibroblasts WI38 normal human fibroblasts were infected with one of two 53BP1 shRNA constructs (sh53BP1 A or sh53BP1 B) or a scrambled shRNA construct for 24 hours and selected in puromycin for 72 hours. (**A**) Representative Western blot and densitometric quantitation showing 53BP1 protein levels as a percentage of scrambled control (set as 1 on y-axis). β-actin levels are included as a loading control. (**B**) Quantitation of micronuclei-positive cells following infection with scrambled or 53BP1 shRNA constructs. Both control, non-damaged cells and cells damaged with 1.5ug/mL neocarzinostatin (NCS) for 2 hours are shown. Data are pooled from three replicates, and at least 400 cells were counted for each condition. Positive cells contain at least one micronucleus. Error bars indicate standard deviation. **P<0.01; ***P<0.001. (**C**) Representative images depicting micronuclei and 53BP1 foci in control and 53BP1-depleted human fibroblasts. Both non-damaged and damaged (NCS) cells were prepared as in (**B**) and stained with DAPI and anti-53BP1 (green). Images showing nuclear structure are depicted for both wild-type fibroblasts and fibroblasts infected with one of two 53BP1 shRNA constructs, and examples of micronuclei are marked with white arrows.

In order to determine whether human cells would display an increase in micronuclei formation similar to mouse cells if the levels of 53BP1 were reduced, WI38 human fibroblasts were infected with one of two shRNA constructs targeting 53BP1 (sh53BP1 A or B), and these constructs led to either a 46% or a 13% reduction in 53BP1 protein levels (Figure 4A). 53BP1-depleted WI38 human fibroblasts were treated with neocarzinostatin to induce DNA damage, and micronuclei were counted as a marker of unresolved DNA damage. The amount of 53BP1 present in human fibroblasts appeared to be associated with the percentage of micronuclei-positive cells (Figure 4B,C). Fibroblasts treated with a scrambled control shRNA construct contained very few micronuclei (1.36% of cells with micronuclei), while fibroblasts with reduced 53BP1 levels had significantly more micronuclei. Interestingly, the level of 53BP1 knockdown correlated with micronuclei, since fibroblasts treated with the shRNA construct producing the 46% knockdown (sh53BP1 A) contained 30.19% micronuclei-positive cells, while fibroblasts exhibiting the 13% knockdown in 53BP1 (sh53BP1 B) contained 13.73% micronuclei-positive cells (Figure 4B,C). Strikingly, the increase in micronuclei-positive cells upon 53BP1 reduction appeared to be independent of DNA damage, since similar numbers of micronuclei were observed in non-damaged cells and the number of micronuclei did not increase further upon DNA damage induction (Figure 4B,C).

### Cell cycle phase influences the response to DNA damage

**Figure 5:**
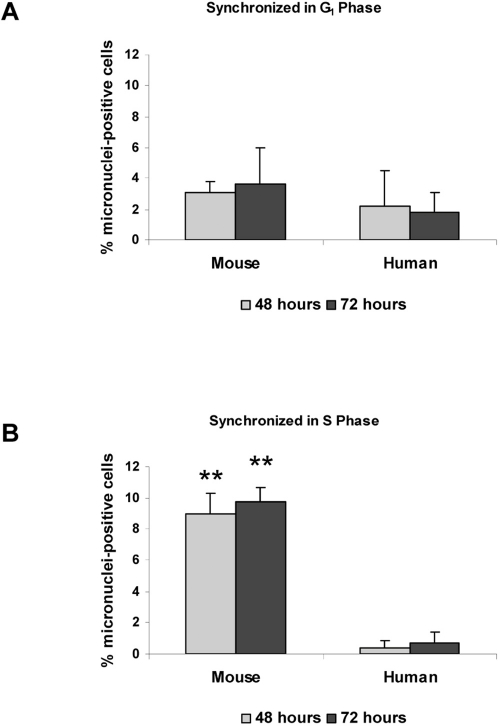
Micronuclei in response to DNA damage are increased during S phase HME mouse fibroblasts and WI38 human fibroblasts were synchronized in G_1_ (**A**) or S phase (**B**). Both cell types were then subjected to DNA damage by treatment with neocarzinostatin (NCS) for two hours. Cells were fixed at 48 hours or 72 hours following NCS treatment, and the number of cells containing at least one micronucleus was counted. Images were analyzed using Slidebook software, and an average micronuclei count was taken from at least 400 cells per treatment and time point. Non-damaged HME and WI38 fibroblasts were included as controls. Differences between mouse and human cells statistically significant at P<0.01 are marked with two asterisks.

Previous work has shown that 53BP1 is involved in transduction of the DNA damage response during several phases of the cell cycle [10,11,12]. As a marker for dividing cells, we examined BrdU uptake in mouse and human cells following damage. We observed that virtually all cells that developed micronuclei were also positive for BrdU, confirming that after damage, progression into mitosis is necessary for micronuclei formation (data not shown). To test the relative importance of cell cycle checkpoints, we arrested both mouse and human fibroblasts in G_1_ phase by serum starvation. Cells were then damaged with neocarzinostatin during G_1_ or released and damaged during S phase. All cells were then allowed to recover from damage for three days. When mouse and human cells were damaged in G_1_ and released, there were relatively low numbers (2-4%) of cells containing micronuclei in both cell types over three days after damage. In contrast, when cells were damaged in S phase then released, 8-10% of mouse cells contained micronuclei by three days, while less than 1% of human cells contained micronuclei (Figure 5B). Additionally, Table 1 shows that when mouse and human cells were damaged in S phase, allowed to recover for 72 hours and then analyzed for their cell cycle position, human cells tended to arrest in G_2_ while mouse cells did not.

**Table 1:** Cell cycle progression following DNA damage in mouse and human cells. Mouse and human cells were damaged in S phase using neocarzinostatin for 1 hour. Cells were then allowed to recover for 72 hours and cell cycle analysis was performed

	G_1_	S	G_2_
Human untreated	68.2	9	22.8
Human neocarzinostatin	37.3	6.1	55.6
Mouse untreated	56.5	3.4	39.6
Mouse neocarzinostatin	57.3	7.4	34.8

### A link between species lifespan and damage resolution

**Figure 6:**
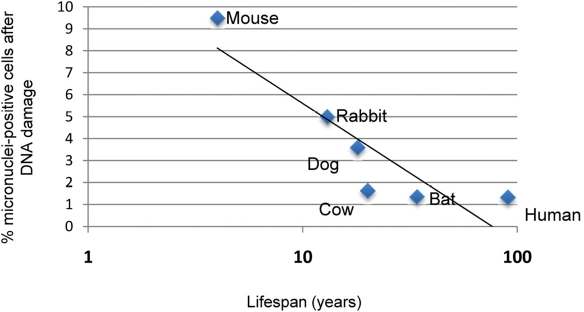
Correlation of micronuclei formation with maximal species lifespan Skin fibroblasts prepared from adult animals of the indicated species under strict standardized culture conditions to ensure uniformity were treated with 1.5 μg/mL neocarzinostatin (NCS) for 2 hours to induce DNA damage. Damaged cells were allowed to recover for 72 hours, after which they were fixed and analyzed for micronucleus formation. Data indicating percentage of cells positive for micronuclei are pooled from two independent experiments. Spearman nonparametric analysis indicates an r value of −0.7982 with a P value of 0.006.

In order to determine whether the genetic instability observed in rodent cells correlated with species lifespan, we analyzed micronucleus formation after DNA damage in fibroblasts from several mammalian species with differing lifespan, which have been established under strictly standardized culture conditions [13]. These species include, in order of increasing maximal species lifespan, mouse, rabbit, dog, little brown bat, bovine, and human. We found that fibroblast cultures from longer-lived species displayed a lower percentage of micronuclei-positive cells when compared with fibroblasts from shorter-lived species (Figure 6). Mouse fibroblasts contained the most micronuclei after damage (9.18% of cells), while human fibroblasts showed the fewest number of micronuclei-positive cells (1.34% of cells), and the percent of cells containing micronuclei demonstrates a negative correlate with lifespan (Spearman correlation coefficient −0.798, P = 0.0006).

## DISCUSSION

The DNA damage response and cell cycle checkpoints are closely linked, and there is substantial overlap of signaling factors in both processes. In addition to the differences our laboratory has observed in Ku80 levels between mouse and human cells [7], we have now demonstrated differences in the accumulation of 53BP1 between these two cell types. Both under normal conditions and in response to treatment with a DNA damaging agent, we find that there is a more robust 53BP1 foci response in human fibroblasts, which display higher numbers of 53BP1 foci over time, compared to mouse fibroblasts. This observation suggests that 53BP1 recruitment may be linked to the differences in genome stability that characterize rodent and human cells. This interpretation is consistent with previous studies that suggest that γH2AX serves to facilitate the accumulation of 53BP1 at damage foci to ensure proper cell cycle arrest [12]. Consistent with this concept, it has previously been shown that 53BP1-deficient mouse fibroblasts cannot maintain G_2_/M arrest as efficiently after damage [14]. Interestingly, it has also been reported that 53BP1 foci may act to shield unresolved DNA lesions occurring as a result of replication stress and that 53BP1 foci may be maintained into subsequent cell cycles; these foci were associated with sites of frequent chromosome breaks such as fragile sites [5]. We noted that mouse cells contain more 53BP1 foci than human cells at baseline (labeled as control in Figure 3C), suggesting that mouse cells are more likely to harbor unresolved DNA lesions than human cells. However, following DNA damage, mouse cells mount a less robust response as evidenced by the reduced number of 53BP1 foci per cell, and presumably this reduced response leads to an increase in the number of cells that produce micronuclei relative to human cells. The link between micronuclei formation and 53BP1 levels is supported by the fact that a reduction in 53BP1 levels in human cells leads to a significant increase in micronuclei even in cells that have not been exposed to DNA damaging agents.

The observation that reduced 53BP1 levels leads to increased micronuclei formation in the absence of DNA damage is somewhat surprising but this result is also consistent with an increase in the number of ultra fine DNA bridges that have been observed in SAOS cells targeted with siRNA directed against 53BP1 [5]. In addition, the use of siRNA against 53BP1 has shown that the protein is required for p53 activation and the induction of the G_2_/M and intra-S phase checkpoints in response to DNA damage [10], and we suspect that the increased accumulation of 53BP1 in human cells is due to the fact that checkpoint activation in response to damaged DNA is more efficient in human cells than in mouse cells. The relationship between micronuclei formation in cultured fibroblasts and species lifespan suggests that the longer-lived species may have a more robust DNA checkpoint control. In fact, a well-established role for 53BP1 is in the transduction of DNA damage signals during most phases of the cell cycle, and 53BP1 colocalizes with phosphorylated H2AX and MDC1 at sites of DNA damage [10,14,15]. It may be that in the absence of exogenous DNA damage 53BP1 acts to activate G_2_ arrest following replication stress. Indeed, 53BP1 has been shown to facilitate NHEJ during S phase to resolve double-strand breaks induced by replication fork stalling [15].

The possibility that 53BP1 action is required during S phase to prevent chromosome damage and micronuclei formation is further supported by the differential response to DNA damage depending upon the cell cycle phase during which damage was induced. When human and mouse fibroblasts were damaged during S phase, mouse cells were more sensitive as demonstrated by increased micronuclei formation, and the G_2_ checkpoint arrest was less stringent as judged by the number of mouse cells that proceeded into G_1_ in the face of DNA damage (Figure 5, Table 1).

In summary, we propose that differences in the accumulation of 53BP1 between mouse and human cells influences genomic stability in both the absence and presence of damaged DNA and forms at least part of the basis for the differences in genome stability observed between these two species.

## MATERIALS AND METHODS

### Cell Culture

WI38 human fetal lung fibroblasts were obtained from Vincent J. Cristofalo. Cells were maintained in Minimum Essential Medium 1X with Earle's salts and L-glutamine (Mediatech, Manassas VA) containing 10% fetal bovine serum, 1X MEM vitamins, 1X MEM amino acids, and penicillin-streptomycin (Mediatech). Fibroblasts were passaged approximately once a week, and population doubling level was determined as previously described [16]. HEK-293T cells for lentiviral production, purchased from ATCC (Manassas VA), were grown in DMEM containing 10% FBS. Fibroblast cultures from mouse (noted as HME), little brown bat, dog, rabbit, and cow were produced and maintained as described previously [13].

### Chemicals

All chemicals used were obtained from Sigma Aldrich (St. Louis, MO) unless otherwise noted.

### Lentiviral Production and Infection

To produce the Ku80 or 53BP1 knockdown WI38 cells, we used a pLKO.1 lentiviral vector containing a short hairpin RNA. The Ku80 shRNA construct was obtained from Sigma Aldrich (MISSION^®^ TRC shRNA TRCN0000039840) and targeted the 5' coding region of the human Ku80 messenger RNA. Two 53BP1 shRNA constructs, also purchased from Sigma Aldrich, targeted the 5' coding region of the human 53BP1 messenger RNA (MISSION^®^ TRC shRNA TRCN0000018866 and TRCN0000018869). Scrambled shRNA plasmid #1864 (Addgene, Cambridge MA) had the following hairpin sequence: CCTAA GGTTA AGTCG CCCTC GCTCT AGCGA GGGCG ACTTA ACCTT AGG [17]. To produce p53-depleted fibroblasts, five p53 shRNA constructs were tested, and the construct providing the most robust knockdown was used (MISSION^®^ TRC shRNA TRCN0000003756) (Supplemental Figure 1). Cells were infected with shRNA viral supernatant along with 10ug/mL polybrene (American Bioanalytical, Natick MA) for 24 hours followed by a 72-hour selection with 2ug/mL puromycin (Mediatech) to eliminate uninfected cells.

### SDS-PAGE and Western blotting

For SDS-PAGE, 30 micrograms of cell extracts in RIPA buffer (50mM Tris, 150mM NaCl, 1% NP-40, 0.1% SDS, 0.5% sodium deoxycholate, 1mM PMSF, 1 ug/mL leupeptin, 1 ug/mL pepstatin) were loaded onto 8% polyacrylamide gels. Gels were transferred to nitrocellulose membranes and blocked for 1 hour in 5% milk in Tris-buffered saline containing 0.1% Tween-20 (TBST). Membranes were incubated with anti-53BP1 (Novus Biologicals, Littleton CO), anti-p53 (Santa Cruz Biotechnologies, Santa Cruz CA), anti-Ku80 (Abcam, Cambridge MA), or anti-β-actin (Sigma Aldrich) in 5% milk TBST overnight with shaking. Membranes were incubated for 1 hour with goat anti-mouse-HRP or goat anti-rabbit-HRP (Millipore, Billerica MA) in 5% milk TBST. Blots were washed in TBST and incubated with ECL Plus chemiluminescent substrate (GE Healthcare BioSciences, Piscataway NJ) for 5 minutes before developing. Densitometry measurements were captured using the ChemiDoc station and software (Biorad, Hercules CA). All densitometric measurements were normalized to loading controls.

### 53BP1 Immunofluorescence

WI38 human and HME mouse fibroblasts were seeded onto glass coverslips and treated with 1.5 ug/mL neocarzinostatin for two hours. Following treatment, cells were fixed immediately or at indicated times after damage in 4% paraformaldehyde for ten minutes and permeabilized in 0.2% Triton X-100 in PBS for one minute. Cells were washed once in PBS and blocked for thirty minutes in 4% BSA in PBS containing Tween-20 (PBST), after which they were incubated in anti-53BP1 in 1% BSA-PBST buffer for 2 hours at 37°C in a humidified chamber. Slides were washed three times in PBST and incubated in AlexaFlour555-conjugated goat anti-rabbit secondary antibody (Invitrogen, Carlsbad CA) in 1% BSA-PBST for one hour to label 53BP1. Cells were washed three times, dehydrated with increasing ethanol concentrations, stained with DAPI, and mounted with Vectashield (Vector Laboratories, Burlingame CA) before visualizing. Images were captured using the Olympus BX61 microscope and Hamamatsu ORCA-ER camera and analyzed using Slidebook software.

### Immunofluorescence for micronuclei

Either asynchronous cells or populations synchronized in G_1_ or S phase were treated with 1.5 ug/mL neocarzinostatin for two hours. To synchronize in G_1_, cells were washed twice in 1X PBS and incubated for 72 hours in serum-free growth medium. Cells were either fixed and stained with DAPI right away or allowed to recover in growth medium for the indicated amounts of time represented in the figures. The cells were fixed in 4% paraformaldehyde for ten minutes and permeabilized in 0.2% Triton X-100 in PBS for one minute. The fixed cells were washed with PBS, dehydrated with 70%, 90%, and 100% ethanol for three minutes each, and mounted in Vectashield containing DAPI. Cells were considered micronucleus-positive if they contained at least one micronucleus. For each time point and treatment, at least 400 cells were scored as positive or negative for micronuclei. Images were captured using the Olympus BX61 microscope and Hamamatsu ORCA-ER camera and analyzed using Slidebook software.

### Statistical Analysis

Statistical significance was measured using one-or two-tailed Student's T test or Fisher's Exact test. Significant changes are indicated with asterisks in the appropriate figures. Regression analysis and Pearson correlation were performed using inSTAT Plus.

## SUPPORTING DATA


