# Supplementary material for: 53BP1 contributes to a robust genomic stability in human fibroblasts
Source: Aging (Albany NY). 2011 Sep 8;3(9):836–45. doi: 10.18632/aging.100381 (PMC3227449; doi:10.18632/aging.100381)
Supplement: Supplementary file 1 [file aging-03-836-s001.pdf]

SUPPLEMENTAL DATA

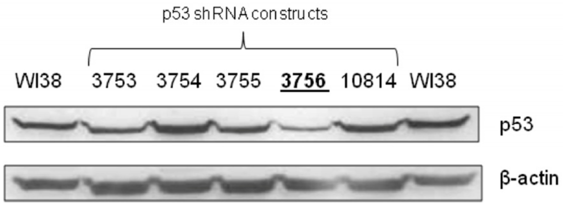

Supplemental Figure 1. Analysis of p53 shRNA constructs in WI38 fibroblasts. WI38 fibroblast cells were infected with one of five human p53 shRNA constructs for 24 hours to determine the construct providing the most robust knockdown. Cells were selected in 2 ug/mL puromycin for 72 hours, and cell lysates were subjected to SDS-PAGE and Western blotting for p53 levels. Construct 3756 (underlined) was chosen for subsequent experiments based on the high degree of p53 knockdown in these cells. β-actin levels are included as a loading control. Two separate lysates from non-infected cells (WI38) were included as additional controls.
